# Forensic Microbiomics and Trace Microbial Evidence: Molecular Innovations for Linking Suspects, Objects, and Environments

**DOI:** 10.3390/microorganisms14081858

**Published:** 2026-08-20

**Authors:** Badal Mavry, Sneha Lohar, Garima Awasthi, Kumud Kant Awasthi, Mahipal Singh Sankhla, Anuj Sharma, Rajeev Kumar, Yogesh Kumar, Ruchi Pathania, Theodoros Varzakas

**Affiliations:** 1 Department of Forensic Science, IILM University, Greater Noida 201306, India; badalmavry08@gmail.com (B.M.); snehalohar011@gmail.com (S.L.); rajeev4n6@gmail.com (R.K.); 2 School of Basic and Applied Sciences, K.R. Mangalam University, Gurugram 122103, India; gariimaa21@gmail.com (G.A.); mahi4n6@gmail.com (M.S.S.); 3 Department of Forensic Science, Parul Institute of Applied Sciences, Parul University, Vadodara 391760, India; anujs0353@gmail.com; 4 Department of Forensic Science, Teerthanker Mahaveer University, Moradabad 244001, India; yogesh.paramedical@tmu.ac.in; 5 Department of Medicinal Chemistry, University of Florida, Gainesville, FL 32610, USA; 6 Department of Food Science and Technology, University of the Peloponnese, 24100 Antikalamos, Greece; t.varzakas@uop.gr

**Keywords:** microbial forensics, trace evidence, case investigation, molecular and biological detection, environment

## Abstract

Microbial forensics has beneficial characteristics for investigating crimes. Law and order are fundamental to any nation and carry significant responsibilities. Failure in this domain may result in wrongful convictions or the guilty remaining free. Key duties include managing criminal cases and addressing threats to public order. While identifying the culprit, establishing their connection to the crime is paramount. Since legal systems require evidence for conviction, law enforcement increasingly relies on advancing scientific methods to meet this need. For example, in this case, after applying conventional techniques like DNA analysis and fingerprinting, a new technique, such as microbial forensics or fingerprints, can be applied. Trace evidence can be in the form of microbial fingerprints, and this evidence can play an important role in solving the crime. Microbial communities are dense and active in living and non-living things and in the environment. The classification and roles of these bacterial populations could be used as markers. The distinction between forensic samples is achievable because there is a diversity of microbial communities found in the human body from one location to another, and even in identical twins. The specific microbiome, which can be found on the skin or in specific body areas, may precisely identify the suspect’s connection with the crime scene for the purposes of human identification. This paper discusses the objects and latest advancements in the science of microbial forensics and their significance within the law enforcement process.

## 1. Introduction

The basis for making links, ensuring justice, and preventing the erroneous imprisonment of the innocent is the comparison analysis on which forensic science relies [1]. Despite the long-term existence of classical DNA profiling as the benchmark, forensic microbiology was still a negligible subspecialty for over a hundred years [2]. The change in approach came at the beginning of the 1990s when it became possible to trace the spread of HIV among dentists’ patients with the help of amplification and sequencing of the virus’s genetic material [3], and rapid development of PCR-based typing of bacteria followed suit [4]. Today, microbial forensics has become an indispensable and prominent tool in the investigative process used by law-related organizations globally, and it is no longer viewed as merely “ancillary” [5,6,7].

Locard’s exchange principle plays an essential role as one of the bases of the modern approach in the field. Each surface in contact with the human body, ranging from fabric to soil and water, creates a virtually infinite trail of trace evidence in the form of continuously shedding microorganisms [8]. The present-day field utilizes these methods of comparison to answer some of the basic investigatory questions—who, what, where, and when did the crime take place? This methodology was first widely employed during bioterrorism investigations in determining the source of biological agents [9,10,11]. Since microbial populations are forms of trace evidence, there is a need to collect them using proper procedures. The unique features of environmental and human microbiomes offer unprecedented diagnostic capabilities. For instance, the specific microbiome, which can be found on the skin or in specific body areas, may precisely identify the suspect’s connection with the crime scene for the purposes of human identification. When dealing with crimes, such as sexual assault, during which traditional DNA analysis might not be applicable, it is useful [12]. Moreover, the environmental microbiomes, obtained from urban areas, transport systems, and soil microorganisms, provide biogeographic data on the geographic source of a particular object, thus establishing a suspect’s relation to that location [13,14]. In addition to the more extensive analysis of microbiomes for cause identification, some indicators of diseases, such as Helicobacter pylori, also establish a person’s relationship with their host organism or environment [15,16].

Although microbial profiling has demonstrated considerable potential for forensic investigations, the evidentiary maturity of different applications varies substantially. Therefore, microbial forensics approaches should be distinguished according to their current level of evidence. Experimental evidence includes findings demonstrated mainly under controlled laboratory, animal-model, or proof-of-concept conditions. Validated forensic applications require analytical validation, reproducibility, appropriate reference populations, quality assurance, and demonstrated performance under relevant casework conditions. Promising but investigational approaches include applications such as individual attribution, microbial geolocation, microbial transfer analysis, and postmortem interval estimation that have shown encouraging results but still require broader inter-laboratory validation, standardized protocols, representative databases, and assessment under real-world forensic conditions. Accordingly, microbial profiles should currently be interpreted according to the strength and limitations of the underlying evidence and should not automatically be considered equivalent to established forensic identification methods.

## 2. Biological Basis and Dynamics of Trace Microbial Evidence

**Figure 1:**
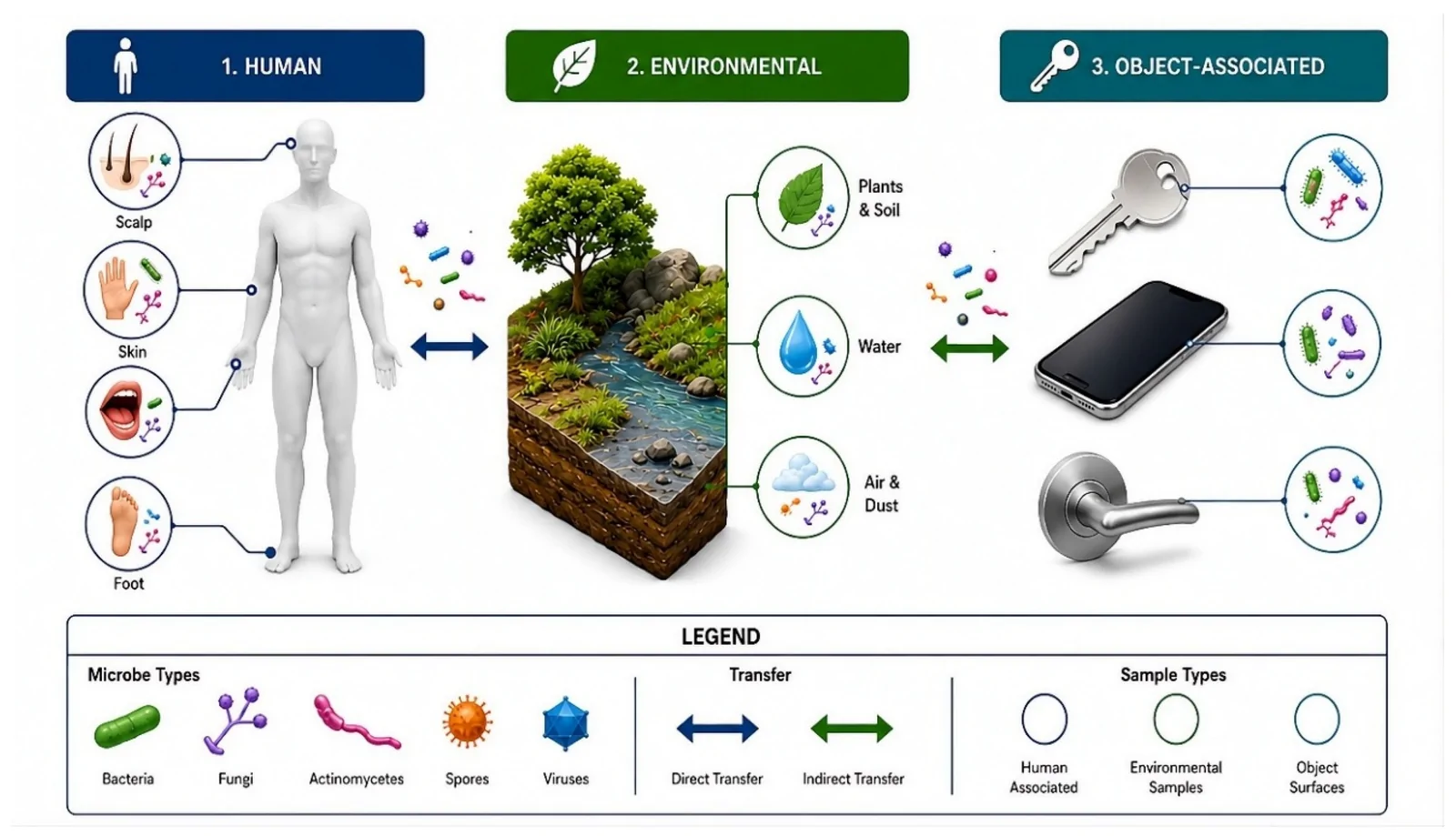
Conceptual overview of trace microbial evidence showing microbial exchange among individuals, biological sites, objects, and environmental sources. The figure illustrates how microbial communities may be transferred through contact and subsequently characterized using molecular and microbiome-based approaches for forensic investigation.

The small communities of bacteria, fungi, and viruses collectively known as the microbiome that get transferred between the host and their environment, including physical objects, are known as trace microbial evidence [17]. Microbial fingerprints can be highly diverse and dependent on context and thus can be used as an entirely novel means of forensics, unlike human DNA, which remains constant among all body cells [18] (Figure 1).

### 2.1. Categories and Diagnostic Accuracy of Microbial Signatures

Robinson et al. [14] indicate that there are three principal sources from which trace microorganisms can be obtained to serve forensic purposes, each of which has its own biological signatures and identification possibilities (Table 1).

### 2.2. Transfer, Persistence, and Temporal Stability

**Figure 2:**
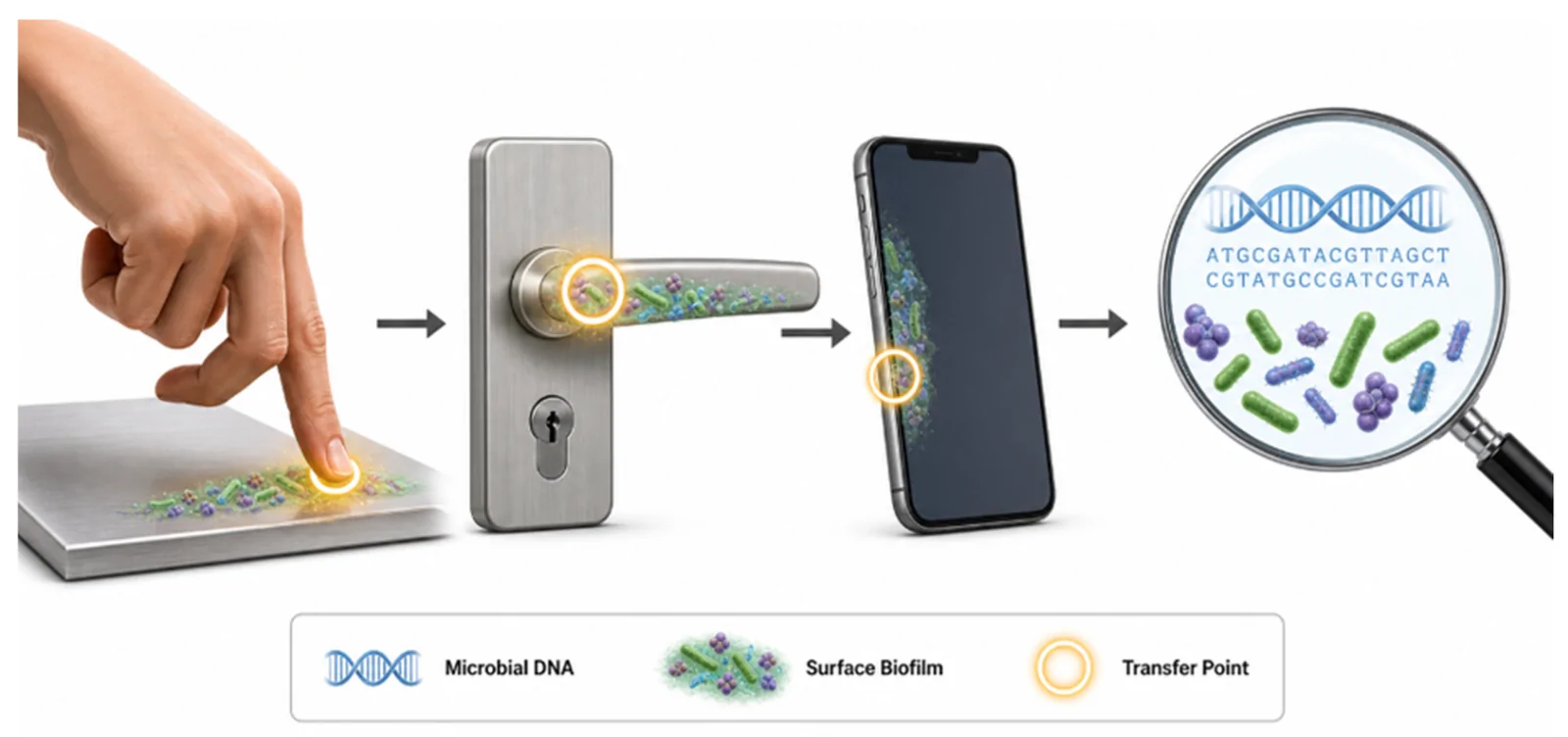
Major biological and environmental factors influencing forensic microbial profiles. Human-associated microbial communities vary according to anatomical site and individual characteristics, whereas environmental microbiomes are influenced by geography, climate, substrate, season, and human activity. These factors can affect microbial persistence, transfer, and forensic interpretation.

The process through which such communities propagate themselves and the duration for which they survive outside their native environment have an important bearing on the forensic value of microbial evidence (Figure 2).

- Methods of Transfer: The processes involved in the deposition of microbes include biological fluid transmission, environmental exposure, and physical contact. Forensic analysis using vaginal microbial signatures has been used to establish the identity of sexual assault offenders when conventional DNA sampling is difficult [26].
- Survival on Surface: Microbes can last remarkably long on contaminated surfaces. Following transfer, some strains of microorganisms, like S. hominis, stay present on human skin for over three days [27]. Successful detection of microbial signatures was recorded after 30 days of exposure to indoor surfaces related to a crime scene [28].
- Temporal Variation: The issue of temporal variation is an important challenge for microbial forensics. Temporal and geographic stability is ensured by the human skin microbiome. However, the oral microbiome is highly susceptible to any sudden changes due to physiological or immune responses [20].

### 2.3. Environmental Factors and Forensic Stability

It is important for researchers to consider biological and environmental factors that influence the evolution of microbe populations so that accurate conclusions about their studies may be made.

- Decomposition and Necrobiome: Some types of bacteria and fungi help predict the progression pattern of the microbial population after death. Differences in environmental or deposition conditions can create problems with interpretation, although studying these events helps in determining the post-mortem interval (PMI) [29,30].
- Geographical and Climatic Variables: The composition of the microbiomes of the environment can depend on the soil, humidity, and climate of the regions. Some types of bacteria present in water environments like Vibrio and Acinetobacter may help link suspects to drowning or other cases [31].
- Collection and Preservation: Avoiding contamination and proper preservation are very important for obtaining accurate profiles from environmental samples. Even though DNA sequences of microbes remain stable even in extremely cold conditions, standardized processes must be used to ensure reproducibility of analysis due to the sample itself (dust, textiles, etc.) [32,33].

## 3. Molecular Approaches in Microbial Detection

### 3.1. DNA-Based Methods (PCR, qPCR)

The key methods used for identifying microbes from forensic samples include PCR and real-time quantitative PCR (qPCR). Traditional PCR on bacterial 16S rRNA genes allows qualitative analysis of the microbiota and identification of individual species. The 16S rRNA gene has adequate genetic variability to enable genus- and species-level identifications due to the presence of conserved and variable regions used in taxonomy [34].

In contrast to conventional PCR, qPCR is more sensitive and can be applied to quantify bacteria and detect diseases in minute quantities. Real-time PCR allows rapid testing that can serve as a first-step screening of forensic samples, focusing on pathogenic bacteria or specific functions (e.g., antibiotic resistance genes) [35]. However, PCR-based techniques also have some significant weaknesses when it comes to the complete assessment of microbial communities. For example, bias in PCR amplification tends to amplify highly abundant microbes while overlooking less abundant members of the microbial community [36]. In addition, systematic biases in the estimation of microbial community composition occur because of the use of primers targeted to certain parts of the genome; the profiles obtained through different primer sets are extremely different [37].

### 3.2. Next-Generation Sequencing (NGS) and Metagenomics

Next-generation sequencing (NGS) technology represents the current gold standard for the evaluation of microbial communities in forensic studies [14]. This technique provides a significantly more detailed characterization compared to culture-based and PCR approaches and allows for parallel analysis of hundreds or even thousands of bacteria and archaea simultaneously. Metagenomic testing, which includes whole-genome sequencing of the complete sample DNA, detects both cultivable and non-cultivable bacteria, thus revealing the entire spectrum of microorganisms in forensic specimens [38]. Amplicon-based 16S rRNA sequencing and long-read approaches are discussed separately below because their analytical objectives, taxonomic resolution, and methodological limitations differ [37]. Full-length sequencing of the 16S rRNA gene is made possible by long-read sequencing technologies, specifically the Oxford Nanopore and PacBio sequencing platforms, which allow for increased taxonomic resolution at the species level. This issue with short-read sequencing, whereby some closely related species cannot be distinguished based on partial gene sequences, can be overcome by full-length sequencing [39]. The forensic identification of microbial samples is therefore improved because of the higher taxonomic resolution provided by full-length sequencing.

### 3.3. 16S rRNA Gene Sequencing

The 16S rRNA gene represents the key genetic marker used for taxonomical profiling of bacteria and archaea in modern microbiology. Evolutionarily conservative sequences of the 16S rRNA gene feature variable regions (V1–V9), which have diverged enough to enable taxonomical profiling at genus and species levels [40]. The alternating conserved and variable regions allow for designing universal primers for PCR amplification that can be applied to all phylogenetically distinct microbial lineages, regardless of preliminary information on the composition of microbial communities [41].

Targeted amplicon sequencing of the 16S rRNA gene’s variable regions (V3–V4 or V4 regions mainly) represents a cost-effective and highly accurate technique for analyzing microbial communities and is applicable for forensic investigation [42,43]. The universal usage of the V4 region through standardized primer sets allows conducting inter-laboratory comparisons of data, contributing to creating forensic databases.

The use of 16S rRNA sequencing in forensic analysis implies several technical limitations to be considered. Operational taxonomical units (OTUs) clustering, previously considered the prevailing technique for identifying bacteria’s species, is being phased out due to the introduction of amplicon sequence variants (ASVs) [44]. The selection of OTU versus ASV greatly impacts estimations of microbial diversity and microbial community composition. Furthermore, the selection of reference taxonomic databases (SILVA, Greengenes, RDP) significantly influences results’ precision and consistency, sometimes leading to contradictory conclusions about species identities in environmental microbes [45].

### 3.4. Whole-Genome Sequencing (WGS)

Whole-genome sequencing (WGS) represents the most accurate approach to microbial identification and detailed characterization in forensic science. WGS allows for accurate species identification, detection of virulence factors and antimicrobial resistance genes, and microbial strain typing that is required for tracing infections and identification of the origin of a pathogen [46]. With the whole-genome sequence, it is possible to perform detailed phylogenic analysis, comparative genomics, and prediction of metabolic functions of microbes. Combining short-read (Illumina) and long-read (Oxford Nanopore or PacBio) sequencing approaches provides more accurate results in comparison with assemblies based on short-read-only data [47]. Improved accuracy in hybrid approaches allows for better discrimination of repeats, mobile DNA, and other complex genetic structures important for forensic microbial strain typing [48].

WGS-based species identification is more accurate than identification based on 16S rRNA gene sequencing. In addition, WGS is useful in complex forensic investigations since it allows for identification of multiple microbial species and virulence factors at once [49].

### 3.5. Bioinformatics Tools and Databases

The analysis of microbial data needs sophisticated computational processing algorithms, verified reference databases, and well-established analytical interpretation frameworks. The analysis workflow related to 16S RNA sequencing analysis (quality control, decontamination and denoising, taxonomic classification, and statistics) greatly influences both community compositions derived by analysis and further forensic interpretation [50]. The different bioinformatics processing pipeline software (QIIME2, Mothur, and MEGAN) generates significantly different results, with QIIME2 showing better performance regarding decreasing the number of false-positive signals and taxonomic misclassifications [51]. Databases of microbial references (SILVA, Greengenes, RDP, NCBI) significantly differ in the extent and completeness of their annotations and taxonomic classifications, especially when considering microorganisms from the environment, which are underrepresented in the collections of clinic-oriented microorganisms [52]. Microbial taxa obtained from environmental samples (soil or water) are less likely to be present in reference clinical databases. The dynamic nature of the metagenomic databases (they are continuously growing due to constant curation and enrichment) suggests that fixed and validated reference databases should be used for the purposes of forensic analyses [52]. The Forensic Microbiome Database (FMD) is an example of the special informatics platform designed specifically for forensic investigations, incorporating microbial data obtained from geographically various locations and from different body parts [53]. Such a database architecture helps to assess possibilities of geographical location identification, individual identifiability, and body fluids determination using comparison analysis methods. Additional dedicated analytical tools can be developed specifically for forensic purposes, such as estimating the post-mortem interval based on microbiome dynamics or machine-learning-based microbial data interpretation [54,55].

The comparative assessment indicates that no single analytical approach is currently sufficient for all forensic microbiological applications. Method selection should therefore consider the biological question, sample quality, analytical sensitivity, required taxonomic resolution, validation status, and intended evidentiary use.

## 4. Advanced Biological and Analytical Techniques

### 4.1. Culturomics and Microbial Isolation

**Figure 3:**
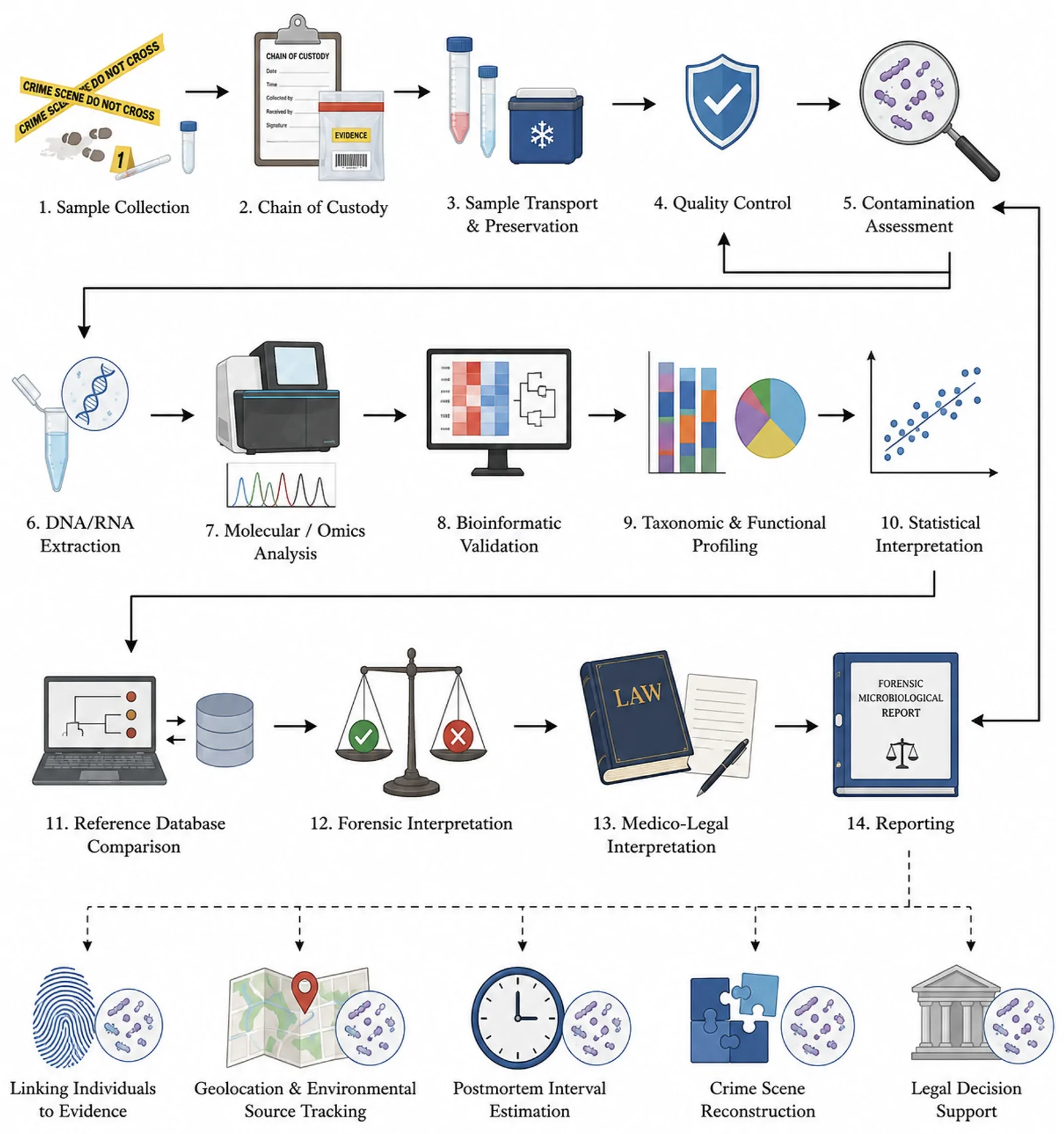
Integrated workflow for forensic microbiological investigation, incorporating sample collection and chain-of-custody procedures, quality control, contamination assessment, molecular and omics-based analysis, bioinformatic validation, statistical interpretation, reference database comparison, and medico-legal reporting.

Modern forensic microbiology heavily favors culture-independent molecular methods; however, traditional culture-based approaches still hold scientific importance. Culturomics, which refers to the growth of microorganisms using different media, environmental conditions, and culture strategies, effectively isolates microorganisms that cannot be identified through molecular methods, yielding viable organisms available for functional studies [55]. Culture-based methods facilitate phenotypic profiling, determination of resistance to antibiotics, and evaluation of biological traits not discernible using sequencing technologies [56] (Figure 3, Table 2).

However, applying culturing methods in forensic microbiota analysis is severely hampered by the obligate lack of cultivability of many microorganisms from the environment and the laboriousness of culture experiments. Nonetheless, the targeted identification of pathogens or clinically important microorganisms can enhance forensic microbiota analysis [17,73]. The process of anaerobic culturing, which is essential for isolating many anaerobes from the environment and from humans, necessitates the presence of specialized laboratory facilities and technical skills in many forensic laboratories [74].

### 4.2. Proteomics and Metabolomics in Forensics

Both proteomic and metabolomic analysis techniques serve as complementary methods to genomic analysis by offering mechanistic insight into the function of the microbial metabolism, its state, and phenotypical properties related to forensics [75]. The protein expression pattern reflects the instantaneous activity status of the microorganism; this way, one can distinguish between active and inactive microorganisms as well as biochemical pathways active in forensic samples [76]. Exometabolomics is defined as the study of both metabolites that are produced and consumed by the microbes, thus providing information on ecological niches as well as functional complementarity in a microbial community. The VOC analysis of biological samples provides additional forensic markers, which can supplement the analysis based on microbial DNA [77,78]. The volatilome includes all volatile compounds produced by humans and is associated with an individual-specific chemical profile potentially useful for forensic purposes. The combined use of proteomics, genomics, and metabolomics in forensic analysis allows for more detailed trace analysis [75,79].

### 4.3. Biosensors and Rapid Detection Methods

Emerging methodologies such as portable biosensors and other rapid identification processes allow for the microbial profiling of biological samples independent of the laboratory setting; Portable DNA extraction instrumentation and quantitative PCR instruments allow for the rapid identification of target microorganisms from environmental samples and increase the rate of analysis from days (or more) to mere hours [80,81]. These techniques allow for the real-time detection of pathogens and preliminary examination of materials from a crime scene‚ producing actionable investigative findings in hours to days [82].

Emerging nano-forensic detection technologies exploit the intrinsic properties of nanoscale materials and nanoengineered devices to improve the analytical sensitivity and specificity of detecting trace DNA and microbial evidence [83]. Nanoparticulate extraction materials‚ engineered biosensor systems‚ and nanochip processing platforms have been developed to ease the extraction‚ amplification‚ and detection of trace DNA samples previously deemed analytically uninformative. Microfluidic systems combined with nano-engineered devices achieve the characterization of DNA in less than one hour with high accuracy [84].

### 4.4. AI and Machine Learning in Microbial Pattern Analysis

Modern-day analysis of forensic microbiomes depends on the use of machine-learning algorithms, which make it possible to detect patterns and classify samples beyond the scope of human capability. Random forest machine-learning models, trained using data from microbiomes, have accuracies of up to 85–95 percent when predicting an individual’s identity, anatomical body site, and geographical location [14]. Deep learning techniques, including transformer architectures, improve the precision of age prediction and phenotype inference from the microbiome profile [20]. Through such advanced machine-learning approaches, complex patterns in the microbial communities can be detected based on their connection with the chronological age, geographical location, and other forensic-relevant variables [85]. The need for interpretable and explainable machine-learning algorithms is paramount in forensics because it facilitates the evaluation of feature importance and validation through separate data sets [86].

## 5. Innovations in Linking Evidence

### 5.1. Linking Suspects to Crime Scenes via Microbial Fingerprints

Microbial fingerprinting may provide supportive information for associating biological traces with individuals or contact events. Experimental studies have demonstrated discriminatory patterns in human-associated microbial communities; however, individual attribution remains investigational because microbial composition varies with anatomical site, time, health, lifestyle, environment, and sampling conditions. Secondary transfer and environmental contamination may further complicate interpretation. Accordingly, microbial fingerprints should not currently be interpreted as definitive individual identifiers without application-specific validation and appropriate statistical support [87,88,89].

The analysis of the microbiological profile of the latent fingerprints provides fresh insights into time-related information that cannot be provided by traditional analyses of ridge pattern analysis methods [90]. The time-dependent succession of microorganisms in the fingerprint samples gives unique “time signatures” which can be used for reconstruction of the time during which the sample was deposited, such as Mycosphaerellaceae and Coxiellaceae being associated with recent deposits while Ruminococcaceae and Beijerinckiaceae being associated with recent contacts with clean hands [87]. Human-associated microbiomes vary with anatomical site, age, health status, diet, medication, hygiene, environment, and time. Therefore, microbial similarity should be supported by appropriate population-level validation and validated statistical frameworks.

### 5.2. Microbial Transfer Between Individuals and Objects

Microbiological transfer between humans and contaminated surfaces proceeds through a predictable ecological process based on contact mechanics and microbial ecology principles. Significant two-way transfers of microbes occur during close body contact involving different groups of microbes, which have signatures distinct from those found in an individual’s reproductive microbiome [91]. The asymmetrical nature of microbial transfer can be explained by physiological differences between reproductive systems and characteristics of microbial populations. Survival of transferred microbiomes occurs on touched surfaces for prolonged periods of time varying depending on substrate type, environmental factors, and microbial group characteristics [92]. Touched areas preserve their own microbial signature associated with the host for long periods of time greater than 72 h and including Staphylococcus hominis associated with integumental skin cells. The persistence of transferred microbial signature allows establishment of connection between individual and contaminated surface but methodological standardization is required before application into forensics practice [93].

### 5.3. Geolocation Using Environmental Microbiomes

Microbial geolocation is influenced by geographical, seasonal, climatic, ecological, agricultural, and anthropogenic factors. Geographical source determination using soil microbiome populations is possible due to significant geographic structuring in soil microbiomes that allows for geographic source identification based on bacterial taxonomic profiles [94]. There is great potential in microbial profiling in predicting geographical origins to specific geographical regions. Such techniques have the potential to provide great utility in tracing evidence to geographical regions in cases where traditional forensic techniques are inadequate [11]. Geographic microbial fingerprints are dynamic due to anthropogenic and natural changes in environmental conditions that alter regional microbiomes. Community shifts in soil microbiomes that occur seasonally, agricultural-to-urban-land-use changes, and community changes caused by climatic changes are some of the environmental factors that affect the dynamic nature of geographic microbial fingerprints. Comprehensive temporal and seasonal databases must be systematically developed as a prerequisite to implementing microbiome fingerprinting for geolocation [95,96]. Robust forensic geolocation requires geographically representative reference databases covering multiple seasons, environmental conditions, and spatial scales. 

### 5.4. Temporal Analysis and Microbial Succession

Succession of the microbial population, which refers to the ordered sequence of changes in microbial populations through time, provides temporal signatures useful for forensics, and among other things, post-mortem interval temporal estimation. Post-mortem microbial succession follows predictable patterns as the result of the process of biological decomposition with some specific bacteria and fungi dominating different decomposition stages [29]. The succession sequence of certain bacterial phyla as well as fungal communities can provide an opportunity for estimation of the post-mortem interval. However, there is considerable variation in terms of successional patterns in relation to the differences in environment, anatomical site, and deposition setting, and this makes it impossible to establish accurate temporal estimates for post-mortem intervals based on microbiome data [97]. Studies showing associations between specific microbes and post-mortem interval under experimental settings have not been able to replicate the results under real-world forensic scenarios (Table 3). The establishment of reference databases detailing microbiome successions in a variety of environments would be necessary before applying this method forensically [98].

The reported time points are study-specific observations or estimates and may represent individual experimental findings or reported time windows rather than means or universally applicable ranges. These values should not be interpreted as precise forensic PMI time points. Microbial succession may vary with decomposition stage, anatomical site, environmental conditions, temperature, humidity, and other factors. Therefore, the reported temporal patterns should be considered supportive indicators and require further validation before routine forensic application.

## 6. Applications in Forensic Investigations

### 6.1. Crime Scene Reconstruction

Crime Scene Investigation (CSI) has become a key specialty in contemporary criminal justice, driven by the application of science, legal criteria, and innovative technologies for analyzing physical and trace evidence for reconstructing crime scenes [7]. Microbiological evidence analysis is increasingly recognized as important in criminal investigations in relation to identification of any individual, their geographical location and to reconstruct the crime scene [99]. Microbial communities can remain on surfaces for extended periods due to their ability to withstand environmental stressors such as humidity, temperature or UV radiation. Since the composition of microbial communities is specific to each person, it is possible to demonstrate the presence of an individual in a particular location, such as a crime scene, and the type of traces left, whether they come from the skin or other body sites [12]. Microbial markers of interest can differentiate specific bacterial species found on various bodily sites/fluids, including the skin, vagina, feces, semen, saliva and hair matrix [28]. This can be helpful in identification and analysis of sexual violence crimes.

Soil microbial samples were analyzed as a function of distance and were found to differ with increasing distance, suggesting that both soil type and geographic location are important factors in microbial community composition [100]. Soils associated with crime scenes exhibit altered bacterial communities due to decomposition of a corpse in the open, resulting in a significant change in available nutrients, biochemistry, and microbiome composition of the local community in addition to that of the corpse [101]. These specific soil changes can also be used as circumstantial evidence of the location of illegal burials [102]. Similar studies related to the determination of immersion time in victims of drowning are also conducted on the basis of changes in the microbial community [103,104].

### 6.2. Post-Mortem Investigations and PMI Estimation

Post-mortem interval is one of the basic factors identified in a forensic investigation, especially in situations where there were no witnesses confirming the circumstances of a person’s death. For this purpose, differential flux in the succession of colonizing microbial communities in major organs has been studied in animal models of pigs and mice [98]. Postmortem microbiology occupies an important interface between microbiology and forensic pathology. Microbial findings obtained after death must be interpreted in relation to the circumstances of death, autopsy findings, anatomical sampling site, histopathological observations, toxicological results, clinical history, and the possibility of bacterial translocation or contamination. The detection of microorganisms after death therefore does not necessarily indicate antemortem infection or establish an infectious cause of death. Contemporary forensic pathology literature emphasizes standardized sampling, appropriate specimen selection, contamination control, and integrated interpretation with conventional medico-legal findings [57,98,105]. To improve the accuracy of PMI estimation, such sequences of exogenous and endogenous bacterial species have been shown to be useful for PMI estimation [106] and to allow assignment to a specific time interval. The first microorganisms microbiologically isolated from the cadaver belong to Staphylococcus species, followed by coliform organisms and various Candida species, whereas the last microorganisms colonizing the cadaver belong to anaerobic species [107]. The postmortem interval is generally divided into following phases (Table 3) [108]. Microbial succession may provide useful temporal information, but PMI estimation based on microbial profiles remains strongly dependent on environmental temperature, humidity, decomposition conditions, anatomical site, cause of death, antimicrobial exposure, microbial biomass, and deposition environment. Reference datasets generated across different environments, seasons, decomposition stages, and population groups are required before microbial PMI estimation can be considered a routinely validated forensic method.

## 7. Challenges and Limitations

### 7.1. Contamination and Sample Integrity

Contamination is a significant interpretative challenge in Postmortem Microbiology (PMM) and can occur at various stages, including sample collection, transportation, and laboratory processing. In forensic settings, sampling frequently occurs under less regulated conditions compared to clinical environments, thereby elevating the risk of introducing exogenous microorganisms [105]. If sampling methods are not done correctly, instruments are reused, or disinfection is not done well, it could lead to false positives that are hard to tell apart from real microbial presence after death or before death. This risk is especially high for samples with low biomass, like blood or cerebrospinal fluid, where even a small amount of contamination can change the meaning of the results [57].

Consensus guidelines emphasize that methodological rigor during sampling is crucial to maintain the diagnostic significance of microbial evidence. Recording sampling conditions, utilizing sterile instruments, and gathering multiple samples for concordance evaluation are essential elements of quality assurance and can be helpful in maintaining the integrity of the microbial evidence [109].

### 7.2. Lack of Standardization and Validation

Quality assurance is a key issue in PMM because differences in sampling, lab procedures, and how results are interpreted can have a big effect on the results. Forensic investigations do not have standard operating procedures that are used by everyone, unlike clinical microbiology. This leads to different practices at different institutions and jurisdictions [57].

Standardization is especially pertinent in the context of molecular techniques and high-throughput sequencing. Different DNA extraction methods, target regions, sequencing platforms, and bioinformatics pipelines can all lead to very different microbial profiles, even when the same samples are looked at [110,111]. In forensic contexts, this level of variability makes it hard to reproduce results and defend them in court. For these reasons, PMM should be part of a structured quality framework that has standard sampling protocols, clear reporting of methods, and structured ways to evaluate results [112].

International working groups, like the European Society of Clinical Microbiology and Infectious Diseases (ESCMID), created a Study Group for Forensic and Post-mortem Microbiology (ESGFOR) and have suggested recommendations based on consensus that deal with important parts of post mortem microbial evidence, such as how to sample, how to choose specimens, and how to record PMI. These guidelines emphasize that methodological rigor is essential for substantive interpretation, irrespective of the analytical technique utilized [11,113].

### 7.3. Medico-Legal Interpretation of Microbial Evidence

From a legal perspective, the absence of standardized protocols, lack of proper laboratory techniques and instruments and validated interpretative frameworks may weaken the admissibility and significance of post-mortem microbiological evidence. Addressing these gaps requires coordinated efforts among forensic pathologists, microbiologists, and legal experts [114].

Microbial species diversity is another big problem that makes it hard to build a national microbial forensics system. Microbial forensics analyses are frequently arduous and information-dense owing to the significant diversity of microbes [115]. Many microbes may be regarded as potential agents in microbial forensics investigations. This intrinsic issue exacerbates the difficulties of species identification and data analysis. The variety of infectious microbes and the fact that most of them are not yet well understood are big problems that a national microbial forensics system will have to deal with in the future. Sufficient biobanking facilities and robust typing systems are needed [7]. Data capture does not only require technical expertise but also well-equipped laboratories that produce genomic, transcriptomic, or proteomic data. Trained scientists and technicians make use of expensive equipment and technologies to produce data needed to understand microbial behavior in biological attacks and disease outbreaks [18].

The legal admissibility of microbial evidence may also depend on jurisdiction-specific standards, including the principles associated with the Daubert and Frye frameworks. Relevant considerations include whether the analytical method has been scientifically tested, subjected to peer review, evaluated for known or potential error rates, supported by appropriate standards and controls, and generally accepted within the relevant scientific community. Because many forensic microbiome applications remain investigational, expert testimony should clearly distinguish validated analytical findings from investigative interpretation and should avoid conclusions that exceed the available scientific validation. Transparent reporting of uncertainty, limitations, validation status, and alternative explanations is therefore essential for responsible medico-legal interpretation.

## 8. Ethical and Privacy Considerations

The human microbiome is a unique biological identifier—a “microbial signature”—that can be attributed to an individual across time, and it plays a significant role in personal identification [116]. Modern metagenomic techniques compromise legitimate privacy expectations by making it easier to anticipate phenotypes and examine microbiota that have been inadvertently deposited on various surfaces [117,118]. Sensitive health information, pharmaceutical regimens, and behavioral patterns may be revealed by microbial remnants [119].

Microbiome data, in contrast to traditional biometric modalities, contains contextual, dynamic information that reflects medical state and personal history [120]. As a result, there are significant privacy lawsuit risks associated with forensic microbiome research, and family cohabitation dynamics may unintentionally divulge collateral information regarding intimate associates [121,122]. Other ethical concerns include the fact that, in contrast to clinical research paradigms, forensic microbiome collecting usually takes place without informed permission. Individual control over signature proliferation is impossible due to the involuntary nature of microbial spread [123]. Permissible analysis procedures for unconsented microbiota are among the crucial ethical issues. In terms of specimen identifiability, subsequent forensic use, and the repatriation of accidental results, institutional biobanks pose significant governance problems [124].

## 9. Future Perspectives and Emerging Trends

### 9.1. Integration with Multi-Omics Approaches and Point-of-Care Tools

The characterization of postmortem microbial communities has become more precise thanks to advancements in sequencing methods. Higher taxonomic resolution and functional insights are provided by shotgun metagenomics and new techniques like 2bRAD-M sequencing, which go beyond conventional 16S rRNA gene sequencing [125]. Shotgun metagenomics is linked to higher expenditures, more computing demands, and an increased risk of environmental contamination, although offering more coverage and functional information (Table 4).

A potential direction for future study is multi-omics techniques, which integrate transcriptomic, metabolomic, and genomic data. However, their usage is currently confined to experimental settings due to their complexity and lack of standardization. More reliable diagnostic techniques are still preferred if they are applied carefully in a forensic context [138].

### 9.2. Emerging Technologies for Forensic Microbiomics

Single-cell sequencing represents another emerging approach that may enable characterization of individual microbial cells within complex communities. This approach could help resolve strain-level heterogeneity and low-abundance microorganisms that may be masked by conventional bulk sequencing. Similarly, spatial transcriptomics and other spatially resolved molecular techniques may provide information regarding the anatomical localization and functional activity of microorganisms within tissues and forensic specimens. Artificial intelligence and machine learning may increasingly support forensic microbiome interpretation by identifying complex microbial patterns, estimating geographical origin, classifying body-site signatures, assisting microbial transfer analysis, and modeling postmortem microbial succession. However, AI-assisted forensic interpretation should incorporate transparent model development, representative training datasets, external validation, estimation of error rates, explainability, and appropriate uncertainty reporting. AI-generated classifications should therefore support rather than replace expert scientific and medico-legal interpretation.

### 9.3. Research Gaps, Future Challenges, and Priorities for Forensic Translation

Despite advances in sequencing, bioinformatics, biosensors, and AI, forensic microbiomics still requires standardized protocols, representative reference databases, and stronger inter-laboratory validation. Key challenges include contamination, microbial transfer, geographical and temporal variability, and uncertainty in individual identification, geolocation, and PMI estimation. Future research should focus on multicentre validation, standardized datasets, reproducible bioinformatics, and real forensic samples to support reliable and appropriately limited casework application.

## 10. Conclusions

One of the most cutting-edge technologies in criminal investigation is microbiological forensic science evidence, which provides a precise and inventive addition—or, in the case of certain evidence, a replacement for conventional forensic procedure. Microorganisms provide detailed biological proof of individual interactions between an organism and its surroundings, making it possible to identify, link evidence, and analyze crime activity across time. The analysis and interpretation of microbiological evidence have been transformed by the integration of recent emerging technologies. This expands forensic potential to incorporate distinct microbial fingerprints on people and their environment in addition to fingerprints and human DNA. Increased application and case studies will improve the uniformity and strength of data gathering, analysis, and interpretation. To acknowledge the legal admissibility, validity of microbiological findings and ethical limitations, regulatory frameworks must also change and a standardized procedure should be incorporated. To place microbiological forensics in the legal system, scientists, forensic specialists, lawmakers, and judges will need to work closely together. Broadly speaking, research, infrastructure, and policy financing will enable a revolutionary leap to a more just and science-oriented system, and microbiological forensic evidence may be the cornerstone of future criminal prosecutions.

## Figures and Tables

**Table 1:** Different categories of microbiome sources and their forensic significance.

Microbiome Category	Key Sources	Forensic Significance and Accuracy	References
Human (Skin, oral)	Teeth, mucosa, nasal canals, palms, forearms, and saliva	Consists of 10^12^ bacteria. Provides a success rate of 95.24% in body site discrimination and 85% in body identification. has been known to consist of 700 or more unique kinds of microorganisms. Highly successful in identifying the perpetrators of crimes such as sexual assaults or bites where traditional DNA methods cannot be used (Leake).	[19,20,21]
Environmental	Soil, water matrices, air/dust	Serves as a finger print of geography. In terms of location, fungal eDNA based on dust can be classified above 82%. The various types of microbes, in particular water bodies, can aid in linking geography in cases of drowning or other crimes involving water.	[22,23]
Object-Associated	Phones, shoes, textiles	A profile of an individual’s journey is achieved through the fusion of environmental exposure (from dirt to shoes) and human contact (from hands to phones). Fabric materials have the potential of carrying bacteria due to their closeness to the body over long periods of time.	[24,25]

**Table 2:** Modern analytical techniques enabling comprehensive microbial characterization and forensic trace evidence analysis.

Analytical Technique	Conventional/Advanced	Sensitivity	Specificity	Limit of Detection (LOD)	Advantages	Disadvantages	References
Culture-Based Methods	Conventional	Low to Moderate	High	Variable; dependent on organism viability	Viable-cell detection; susceptibility testing; low cost.	Low postmortem sensitivity; antibiotic and contamination effects; misses fastidious organisms.	[57,58]
Traditional PCR (16S rRNA)	Conventional	Moderate	Moderate to High	~10^1^–10^2^ copies	Rapid targeted detection; species-level identification.	Amplification bias; incomplete community profiling.	[59,60]
Real-Time PCR (qPCR)	Conventional	High	High	~10^1^ copies	Sensitive; quantitative; rapid.	Target-specific; primer bias; limited diversity profiling.	[57,61]
Targeted PCR Assays	Advanced	High	High	~10^1^–10^2^ copies	Sensitive; rapid pathogen detection.	Target-specific; cannot confirm viability; contamination risk.	[57,62]
16S rRNA Gene Amplicon Sequencing	Advanced	Moderate to High	Moderate	~10^1^–10^2^ copies	Culture-independent profiling; cost-effective; scalable.	Limited taxonomic resolution; primer bias; limited functional information.	[59,63]
Shotgun Metagenomics	Advanced	High	High	~10^2^–10^3^ copies (variable)	Broad taxonomic and functional profiling; genome recovery.	Costly; computationally demanding; contamination and host-DNA effects.	[59,64,65]
Whole Genome Sequencing (WGS)	Advanced	Very High	Very High	~10^2^–10^3^ copies	High-resolution identification; strain typing; comparative genomics.	Costly; computationally intensive; specialized expertise required.	[18,66]
Metatranscriptomics	Advanced	Moderate to High	Moderate	Variable	Profiles microbial activity and gene expression.	RNA instability; host-RNA contamination; complex analysis.	[59,67]
Metaproteomics	Advanced	Moderate	Moderate	Variable (ng-pg range)	Taxonomic and functional profiling; community interaction analysis.	Low-abundance protein detection; database and reproducibility limitations.	[59,68]
Metabolomics	Advanced	Moderate	Moderate	10–100 nM (LC-MS); >1 mM (NMR)	Profiles microbial metabolites; biomarker discovery.	Incomplete databases; many unidentified metabolites; complex integration.	[59,69]
Nano-Forensic Detection Technologies	Advanced	Very High	Very High	Reduced to trace levels	High sensitivity; enhanced biosensing.	Limited standardization; costly; largely experimental.	[18,70,71]
Portable Point-of-Care (POC) Devices	Advanced	High	High	Very low (down to <10 copies)	Rapid; portable; field-deployable; sensitive.	Requires validation; reduced specificity in complex samples.	[18]
AI and Machine Learning Models	Advanced	Variable	Variable	Not applicable	Complex-pattern recognition; high-dimensional data analysis.	Limited forensic validation; interpretability and generalizability issues.	[57,72]

**Table 3:** Comparative evaluation of molecular, sequencing, biosensor, and artificial intelligence approaches for forensic microbiological analysis.

Method	Analytical Principle	Advantages	Limitations	Analytical Performance	Major Forensic Applications
PCR	Target-specific DNA amplification	Rapid, sensitive, relatively inexpensive	Target dependent; contamination risk; limited multiplexing	High sensitivity for defined targets	Pathogen detection, targeted microbial identification
qPCR	Real-time quantitative DNA amplification	Rapid quantification; high sensitivity; low biomass detection	Requires predefined targets; primer bias	High sensitivity and quantitative capability	Screening, microbial abundance, targeted detection
16S rRNA sequencing	Amplicon sequencing of bacterial/archaeal 16S regions	Cost-effective community profiling	Primer bias; limited species-level resolution; database dependence	Moderate to high taxonomic resolution	Microbial profiling, body-site discrimination, environmental comparison
Metagenomics	Untargeted sequencing of microbial DNA	Broad community and functional characterization	Expensive; computationally demanding; contamination and database limitations	High taxonomic and functional information	Source attribution, environmental profiling, microbial community analysis
WGS	Complete microbial genome sequencing	High strain-level resolution and genomic characterization	Requires sufficient DNA; cost and computational requirements	Very high genomic resolution	Strain typing, microbial source investigation, pathogen characterization
Biosensors	Molecular/biological recognition with sensor-based detection	Rapid, portable, potentially field deployable	Target dependent; validation and matrix effects	Rapid targeted detection	Crime-scene screening and preliminary microbial detection
AI-assisted methods	Machine learning and pattern recognition	Handles complex datasets; classification and prediction	Training bias, explainability, overfitting, database dependence	Model dependent	Pattern classification, geolocation, microbial attribution, PMI research

**Table 4:** Necrobiome-associated bacterial taxa, anatomical distribution, and characteristic temporal succession patterns used as biomarkers for post-mortem interval estimation.

S.No.	Necrobiome Species/Taxa Name	Anatomical Location	Estimation of Time Since Death (PMI)	References
1	*Staphylococcus aureus*	Skin	Peaks at 5–7 days post-mortem, then decreases until it is no longer detectable at 30 days.	[30,126,127]
2	*Ignatzschineria* spp.	Body/Skin	Common during the wettest phases (bloat and purge stages) and decreases during the dry stage.	[128,129]
3	*Wohlfahrtiimonas* spp.	Body	Common during the bloat and purge stages, often associated with myiasis.	[128,130]
4	*Acinetobacter* spp.	Body/Brain	Common after tissue dehydration and skeletonization on the body; in the brain, abundance significantly increases around 8 h.	[30,131]
5	*Streptococcus* spp.	Eyes/Mouth	Indicates an early PMI, acting as a potential biomarker for the first 48 h (<24 h to 48 h).	[132,133,134]
6	*Pseudomonas* spp.	Mouth	Detected during the pre-bloat stage but absent by the end-bloat stage.	[132,135]
7	*Haemophilus parainfluenzae*	Mouth	Acts as a potential bioindicator for a PMI of <48 h.	[132]
8	*Clostridium tetani*	Gut	Appears around day 7, remains until day 15, and then decreases.	[132,136]
9	*Enterococcus faecalis*	Rectum/Gut	Appears around day 2, and its relative abundance increases significantly after 5 days.	[130]
10	*Proteus mirabilis*	Rectum	Relative abundance increases after 5 days post-mortem.	[128,130]
11	*Clostridium sporogenes*	Rectum	Highly abundant in the early post-mortem period, specifically before 1 day.	[30,132]
12	Planococcaceae (Family)	Skin	Dominates the skin microbiota consistently from the beginning to the end of the decomposition process.	[128,135]
13	Pseudomonadaceae (Family)	Oral Cavity/Soil Interface	Predominant during the early (active decay) phase of decomposition.	[128]
14	Lactobacillus	Internal Organs (MLNs, liver, spleen)	Predominant in early PMI (e.g., 3 to 12 h) and declines exponentially as PMI increases.	[134]
15	Bacteroides	Proximal large intestine	Declines exponentially with increasing PMI.	[132]
16	Parabacteroides	Proximal large intestine	Abundance decreases significantly over time as decomposition progresses.	[136]
17	Anaerosphaera	Proximal large intestine	Increases significantly over time toward a common decay community.	[128,129]
18	Rothia	Mouth/Internal Organs	Dominant taxa in a <24 h time window; found specifically in internal organs of male cadavers.	[132,134]
19	Enterobacteriaceae (Family)	Internal Organs/Mouth	Translocates to extra-intestinal compartments within 5 min of death; shows increased relative abundance as PMI increases.	[132]
20	Bacillaceae (Family)	Mouth	Principal representatives of Firmicutes from the bloat stage to advanced decay.	[135]
21	Clostridiales (Order)	Mouth	Principal representatives of Firmicutes from the bloat stage to advanced decay.	[128,134]
22	α-Proteobacteria	Skeletal Remains (Ribs)	Abundance increases for each successive decay stage from partially skeletonized to dry remains.	[137]
23	γ-Proteobacteria	Skeletal Remains (Ribs)	Abundance decreases for each successive decay stage from partially skeletonized to dry remains.	[137]
24	Actinobacteria/Acidobacteria	Skeletal Remains (Ribs)	Becomes dominant in the dry remains stage.	[137]

## Data Availability

The original contributions presented in this study are included in the article. Further inquiries can be directed to the corresponding authors.
